# Unexpected Onset of Severe Hypothyroidism Following a Thyroid Technetium Scan in a Young Patient With Graves' Disease: A Case Report

**DOI:** 10.7759/cureus.87460

**Published:** 2025-07-07

**Authors:** Hasan F Jamal, Sayed Mohamed Ebrahim, Abdulla Ahmed Alsaie, Reem A Hubail, Ali H Ali

**Affiliations:** 1 Endocrinology, Diabetes and Metabolism, Salmaniya Medical Complex, Manama, BHR; 2 Internal Medicine, Salmaniya Medical Complex, Manama, BHR

**Keywords:** case report, graves' disease, thyroid function tests (tfts), thyroid-stimulating hormone (tsh), hashimoto’s thyroiditis

## Abstract

This is a case report of a 20-year-old Bahraini man who presented for a regular check-up. Upon further questioning, he complained of difficulty gaining weight. Initial laboratory results indicated Graves’ disease (thyroid-stimulating hormone (TSH), 0.02 mIU/L; free T4, 29 pmol/L), which was confirmed by homogeneous diffuse uptake on thyroid scintigraphy. Shortly after the iodine-based scan, the patient developed tremors, palpitations, and dizziness. A thyroid function test (TFT) revealed an abrupt transition to severe hypothyroidism (TSH, 141.76 mIU/L; free T4, 3.3 pmol/L).

This shift could be explained by iodine-induced thyroid dysfunction in the context of underlying autoimmunity or other possible mechanisms, such as the Wolff‒Chaikoff effect, iodine-induced thyroiditis, or autoimmune switching leading to biochemical hypothyroidism. The article addresses a rare phenomenon and highlights the need for careful monitoring after iodine-based imaging in patients with autoimmune thyroid disease.

## Introduction

Graves’ disease and Hashimoto’s thyroiditis are the two most prevalent autoimmune disorders of the thyroid [1]. As noted by the National Institute of Diabetes and Digestive and Kidney Diseases (NIDDK), their pathogenesis involves a multifactorial interplay of genetic predisposition and environmental triggers, with family history serving as a significant risk factor [2, 3]. Graves’ disease is characterized by the presence of thyroid-stimulating immunoglobulins (TSIs), resulting in hyperthyroidism [2]. A radioactive iodine (RAI) scan typically reveals diffuse uptake. Management options include anti-thyroid agents such as methimazole, RAI ablation, and, in selected cases, thyroid surgery [4, 5].

Hashimoto’s thyroiditis is an autoimmune condition characterized by chronic lymphocytic infiltration of the thyroid, resulting in progressive glandular destruction and hypothyroidism (elevated thyroid-stimulating hormone (TSH) and reduced free T4 and T3). It is characterized by the presence of anti-thyroid peroxidase (anti-TPO) and thyroglobulin antibodies (TgAbs) [6, 7]. RAI uptake is diffusely reduced, and treatment involves lifelong levothyroxine replacement [7]. Technetium-99m (Tc-99m) pertechnetate is taken up by the thyroid but not organified [8]. Thus, it does not interfere with thyroid hormone synthesis or affect serum TSH, T3, or T4 levels.

The following case presented a rare sequence of events, marked by an abrupt transition from Graves' hyperthyroidism to hypothyroidism soon after a diagnostic iodine scan.

## Case presentation

A 20-year-old Bahraini male with pre-existing medical conditions presented to a healthcare center with a persistent difficulty in gaining weight despite a high caloric intake exceeding 3000 kcal per day. The significant family history included hypothyroidism and thyroid cancer. The evaluation was initially focused on general health assessment; however, thyroid function tests (TFTs) were included as part of the comprehensive workup.

Initial laboratory findings revealed a suppressed TSH level of 0.02 mIU/L and a T4 level of 29 pmol/L, which suggested hyperthyroidism (Table 1) as part of the investigations. The patient was referred to an endocrinologist for further evaluation.

**Table 1:** Thyroid function test illustrating the trend of transition through various stages starting from Graves’ disease hyperthyroidism, followed by a sudden onset of hypothyroidism, and eventually gradual stabilization to euthyroidism. TSH: thyroid-stimulating hormone; anti-TPO: anti-thyroid peroxidase antibodies; anti-Tg: anti-thyroglobulin antibodies

Date	TSH (mIU/L)	Free T4 (pmol/L)	Anti-TPO (IU/mL)	Anti-Tg (IU/mL)	Interpretation
Nov 2023	0.02	29.0	>1300	2.0	Graves’ disease
Jan 2024	141.76	3.3	–	–	Severe hypothyroidism
Mar 2024	11.77	13.7	–	–	Gradual stabilization
Jul 2024	3.24	–	–	–	Gradual stabilization
Reference range	0.55-4.78	10.7-18.4	≤28	≤100	–

At the endocrine consultation, despite mild palpitations, the patient reported no classic hyperthyroid symptoms, such as heat intolerance, tremors, or gastrointestinal disturbances. On physical examination, a mild, diffuse goiter was noted without tenderness or nodules. Thyroid scintigraphy was requested to further evaluate thyroid activity.

**Figure 1:**
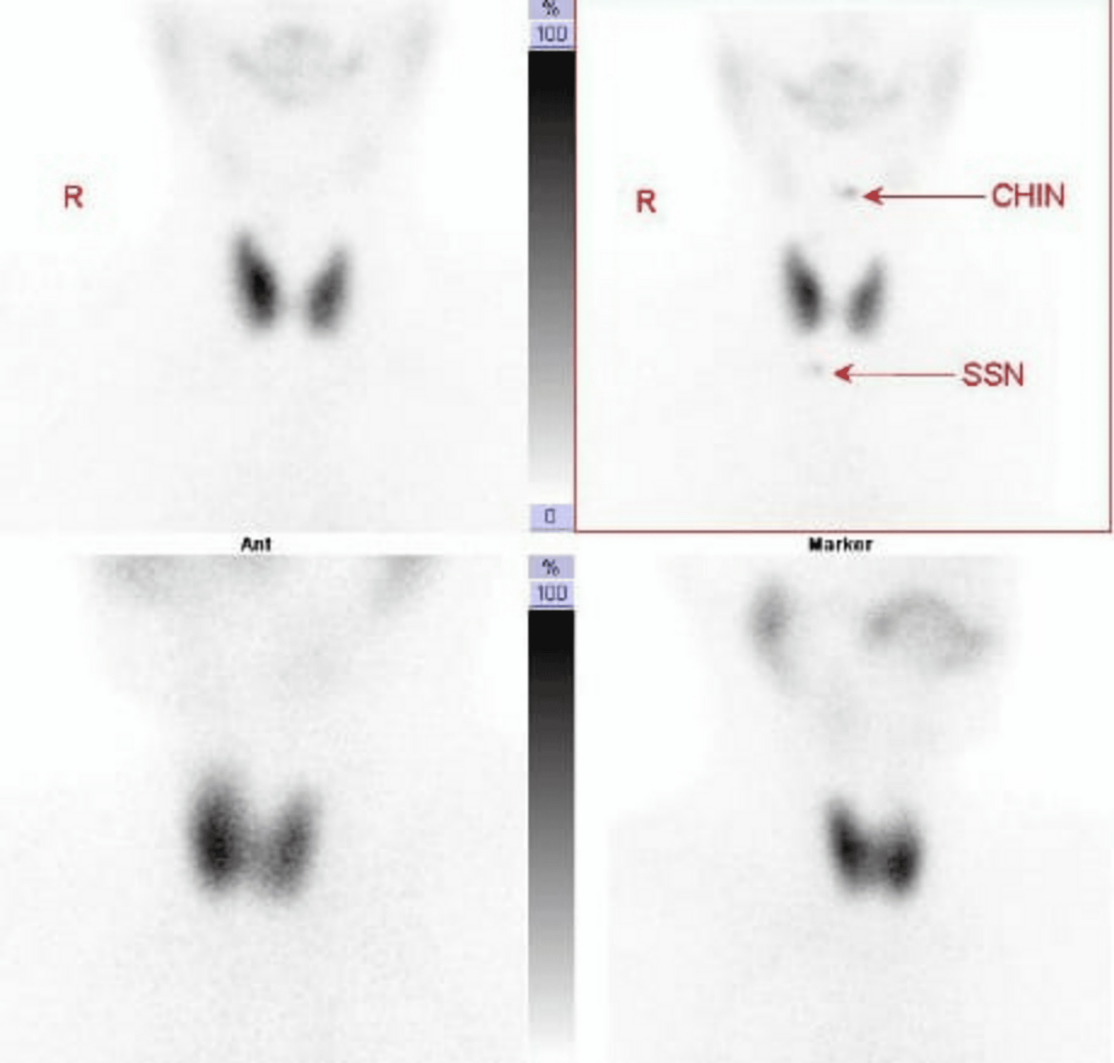
The thyroid scan showed that the thyroid gland appeared normal in size, with no gross retrosternal extension. It demonstrated homogeneously increased tracer distribution, with associated reduced salivary gland and background activity. No definite focal area of increased or decreased tracer uptake was noted to suggest a hot or cold nodule. Uptake at 20 minutes post-injection was 6.5% (normal uptake: 0.4–4%). Scintigraphic findings suggest diffuse toxic thyroid. CHIN: chin marker, SSN: suprasternal notch

Following the administration of iodine for the thyroid scan, the patient experienced pronounced tremors in the hands and legs, some palpitations, and dizziness. These symptoms lasted for approximately 30 minutes and gradually resolved on their own. He proceeded with the scan, which was completed without further complications. Thyroid imaging revealed a homogeneous, diffuse increase in RAI uptake in both lobes of the thyroid gland, which is consistent with Graves' disease (Figure 1). Thyroid imaging was performed following intravenous administration of 187 MBq (5 mci) Tc-99m pertechnetate.

In a surprising clinical turn, repeat TFTs 15 days after the scan showed a TSH level of 141.76 mIU/L, indicating a transition to overt hypothyroidism (Table 1). He was advised to repeat the test to confirm the diagnosis, and the results from external laboratories confirmed persistent hypothyroidism.

Initially, 100 mcg of levothyroxine daily was prescribed, and thyroid function was monitored monthly. The TSH levels at follow-up were 11.77, 3.24, and 3.59 mIU/L, respectively, indicating a slow trend toward normalization (Table 1). After stabilization, the levothyroxine dose was reduced to 50 mcg. Throughout the process, the patient maintained an active lifestyle and reported feeling better overall.

## Discussion

This case report describes a previously healthy 20-year-old man who presented with a rare and abrupt transition from a state of hyperthyroidism, which was due to Graves’ disease, to full-blown hypothyroidism shortly following receiving diagnostic RAI for thyroid scintigraphy. The sequential association between diagnostic RAI administration and the sudden onset of hypothyroidism raises clinical questions regarding iodine-induced thyroid dysfunction, particularly in the context of autoimmune thyroid disease.

Graves’ disease is classically characterized by suppressed TSH levels, elevated free thyroid hormones, and diffuse increased uptake on RAI scintigraphy [2]. Our patient initially had a clinical and biochemical picture consistent with Graves' disease, with symptoms of inability to gain weight. The presence of a mild goiter and elevated RAI uptake confirmed the diagnosis. However, the dramatic shift to severe hypothyroidism shortly after exposure to RAI is an atypical and notable presentation.

Graves’ disease is primarily mediated by thyroid-stimulating immunoglobulins (TSAbs), which bind to and activate the thyroid-stimulating hormone receptor (TSHR). However, a limited number of patients can develop thyroid-blocking antibodies (TSBAbs), resulting in hypothyroidism, a phenomenon termed autoimmune antibody switching [9, 10].

Oueslati et al. reported a patient who developed Hashimoto's thyroiditis after receiving radiation therapy for the thyrotoxicosis of Graves' disease. This patient’s condition was characterized by the development of hypothyroidism and elevated anti-TPO antibodies post-radioiodine therapy [11]. Although the iodine used in this case was therapeutic rather than diagnostic, the pattern of events aligns with the hypothesis that exposure to iodine may trigger an autoimmune phase shift.

Some proposed mechanisms are speculative due to the lack of sequential antibody titers or thyroid ultrasound data. One of these effects is the Wolff-Chaikoff effect, an autoregulatory phenomenon in which high iodine levels temporarily inhibit thyroid hormone synthesis [12,13]. In the Wolff-Chaikoff effect, there is a suppression of sodium/iodide symporter (NIS) and thyroid peroxidase; however, most individuals escape this effect within two days, and failure to escape, especially those with underlying autoimmunity (anti-TPO+), may lead to hypothyroidism [14].

Additionally, the patient's sudden transition to a hypothyroid state may suggest iodine-induced thyroiditis; this could have potentially been triggered by oxidative stress and inflammatory changes following the patient's exposure to the RAI. This has been documented in the literature, particularly in patients with an underlying autoimmune predisposition, in which the sudden influx of the RAI can provoke thyroid cell damage and hormone release followed by a hypothyroid phase. [15]. In this case, it is plausible that the patient had evolving or subclinical autoimmune thyroiditis that was exacerbated by iodine exposure.

Graves’ disease can progress rapidly within months. In our case, Graves’ disease abruptly transitioned to overt hypothyroidism, most likely accelerated by iodine exposure in the presence of positive anti-TPO antibodies, which is in line with the description by Effraimidis et al. [16, 17].

Anti-TPO antibodies can be present in Graves’ disease and may predict the eventual development of hypothyroidism. Furthermore, elevated anti-TPO levels are associated with a poorer prognosis and an increased risk of glandular failure, which explains the abrupt transition to biochemical hypothyroidism in our patient. They also highlighted that iodine exposure may potentiate autoimmune thyroid destruction in anti-TPO-positive individuals, aligning with our case, where a Tc-99m scan likely contributed to the development of hypothyroidism [16, 17].

Although some retrospective studies have linked stress to the development of Graves’ disease or thyroid autoimmunity [18-20], evidence from Effraimidis et al. [17] indicates that anti-TPO positivity and autoimmune thyroid progression are not associated with stress. These findings support the conclusion that the hypothyroid shift observed in our patient was most likely driven by iodine exposure and underlying autoimmunity rather than psychosocial factors.

Alternative causes of hyperthyroidism, such as thyroiditis and toxic nodules, were mainly excluded based on thyroid scintigraphy. The elevated RAI uptake observed in our case makes thyroiditis unlikely, as it usually shows low or absent uptake. In contrast, toxic adenoma typically shows focal uptake in a single area, and toxic multinodular goiter shows multiple areas of increased uptake [5]. In addition to the thyroid scan findings, the presence of clinical features, elevated anti-TPO antibodies, and initial TFTs supports the diagnosis of Graves’ disease and reinforces an autoimmune process rather than nodular pathology.

There are a number of limitations encountered in this case report that should be acknowledged. First, the lack of testing for TSIs was due to its unavailability in our facility. However, the diagnosis of Graves' disease was strongly supported by clinical manifestations, TFTs, and imaging findings. The lack of TSIs indicates difficulty in confirming that the definitive diagnosis was Graves’ disease and explains the occurrence of autoimmune switching between TSIs and TSBAbs.

## Conclusions

This report presents a rare but plausible thyroid outcome that adds meaningful clinical awareness to post-scan surveillance in autoimmune thyroid cases. The clinical pattern clearly points to an underlying autoimmune mechanism, which may be worsened by the Wolff‒Chaikoff effect, iodine-induced thyroiditis, or a change in the balance of stimulating to blocking TSH receptor antibodies. However, causality cannot be definitively established from a single case. This case highlights the importance of recognizing the potential for iodine exposure to precipitate or unmask autoimmune thyroid dysfunction, which is particularly important in patients with pre-existing thyroid autoimmunity. Our case suggests that in high-risk individuals, anti-TPO antibodies can be used as a valuable screening tool to guide the decision-making process and optimize the timing between diagnostic evaluation, initiation of treatment, and thyroid scan. Routine monitoring of thyroid function is essential following the administration of RAI to monitor and ensure timely diagnosis and management.
